# T-cell receptor anti-CMV CDR3s from bone marrow samples are associated with a worse overall survival for recurrent acute myeloid leukemia

**DOI:** 10.1016/j.lrr.2026.100580

**Published:** 2026-03-14

**Authors:** Srijit Paul, Michael T. Aboujaoude, Vayda R. Barker, Tabitha R. Hudock, Arpan Sahoo, George Blanck

**Affiliations:** a Department of Molecular Medicine Morsani College of Medicine, University of South Florida, Tampa, FL 33612, USA; b Department of Immunology, Moffitt Cancer Center and Research Institute, Tampa, FL 33612, USA

**Keywords:** Acute myeloid leukemia, Anti-viral complementarity determining region-3, T-cell receptor, Co-morbidities, CMV

## Abstract

We assessed the relationship of AML patient outcomes and T-cell receptor (TCR) recombination reads representing known anti-viral TCR sequences. TCR complementary determining region-3′s (CDR3s) were obtained from the pediatric TARGET-AML dataset, and the TCR CDR3s representing bone marrow samples of primary and recurrent disease were matched to anti-viral TCR CDR3s. The patients representing the presence of the anti-viral CDR3s were then assessed for overall survival (OS). Patients with anti-CMV TCR CDR3s from bone marrow samples representing recurrent AML showed a significant decrease in OS probability. Decreases in OS probabilities were also observed for patients with anti-Epstein Barr Virus (EBV) TCR CDR3s obtained from recurrent AML, bone marrow samples. Overall, this study raises the question of whether there is a relationship between viral infections, or the basic impact of anti-viral inflammation, and outcomes in pediatric AML?

## 1 Introduction

Pediatric Acute Myeloid Leukemia (AML) is a rare hematological cancer characterized by rapid growth of malignant hematopoietic stem cells. Unlike the more prevalent Acute Lymphoblastic Leukemia (ALL), AML presents with markedly worse overall survival (OS) and a 30 % relapse rate in children [1]. The presence of opportunistic viruses in immunocompromised individuals, such as AML patients, is well documented, and in many instances, the viral infections likely have a negative impact on outcomes [2]. However, paradoxically, CMV has also been implicated in an immune-protective role and may reduce relapse of AML and reduce other negative outcomes of the cancer [3,4].

The complementary determining region-3 (CDR3) of T-cell receptors (TCRs) provides insight into the variability of amino acids (AAs) that make contact with antigen epitopes. Over several decades, the anti-viral CDR3 AA sequences have been catalogued and annotated in databases [5,6]. This large set of anti-viral CDR3 AA sequences provides an opportunity to assess, in a novel, bioinformatics-oriented manner, whether TCR CDR3s obtained from patient tissues match known anti-viral CDR3s. Also, there is the opportunity to inform the discovery of such anti-viral CDR3s with clinical or related data, such as viral DNA detection in patients. This paradigm has been informative in several cancer settings, most importantly neuroblastoma [[7], [8], [9]]. Thus, in an attempt to further understand and clarify the impact of viral infections in AML, the relationship between the detection of anti-viral CDR3s and patient outcomes was determined for AML.

## 2 Methods

### 2.1 Mining of the TCR recombination reads

TCR recombination reads were mined from the Therapeutically Applicable Research to Generate Effective Treatments (TARGET; phs000218) AML dataset. The controlled access, RNAseq files for the TARGET dataset were accessed according to National Institutes of Health (NIH) database of Genotypes and Phenotypes (dbGaP) project approval number 16,405. The mining software pipeline and the translation of CDR3 AA sequences are described in detail in refs [[10], [11], [12]], and the latest version of the software is freely available at https://github.com/arpansahoo/vdj. The complete TRA and TRB recombination read outputs are in supporting online material (SOM) Table S1. Samples for preparation of RNAseq files were collected from pediatric patients with AML enrolled in Children’s Oncology Group trials at time of diagnosis and in the instance of relapse in disease (Table S2; https://www.cbioportal.org/study/clinicalData?id=aml_target_2018_pub).

### 2.2 Matching TARGET-AML TCR CDR3s with anti-viral CDR3s

R v4.2.2 with packages contained within ‘tidyverse’ v2.0.0 were utilized to identify TARGET-AML cases with exact TCR CDR3 AA matches to anti-viral TCR CDR3 sequences. Anti-viral TCR CDR3 AA sequences were sourced from VDJdb (https://vdjdb.cdr3.net/). Selected TARGET-AML cases were then associated with clinical data sourced from cBioPortal (https://cbioportal.org/) to provide associated demographic and disease state data.

### 2.3 Survival analyses

OS probability was calculated using R v4.2.2 packages ‘survival’ v3.5–7 and ‘survminer’ v0.4.9. Kaplan Meier (KM) curves were generated comparing selected cases to remaining cases available and a log-rank p-value <0.05 was considered statistically significant.

### 2.4 Multivariate analyses

Multivariate Cox regression analysis was performed using R studio. TRA/TRB recombination reads from recurrent bone marrow representing matching anti-CMV CDR3s, sex, race category, and risk group were defined as categorical variables. Diagnosis age, white blood cell count (WBC), and peripheral blast percentage were treated as continuous variables. Case IDs missing one or more variables were excluded. Further details can be found in refs [13,14].

## 3 Results

**Fig. 1:**
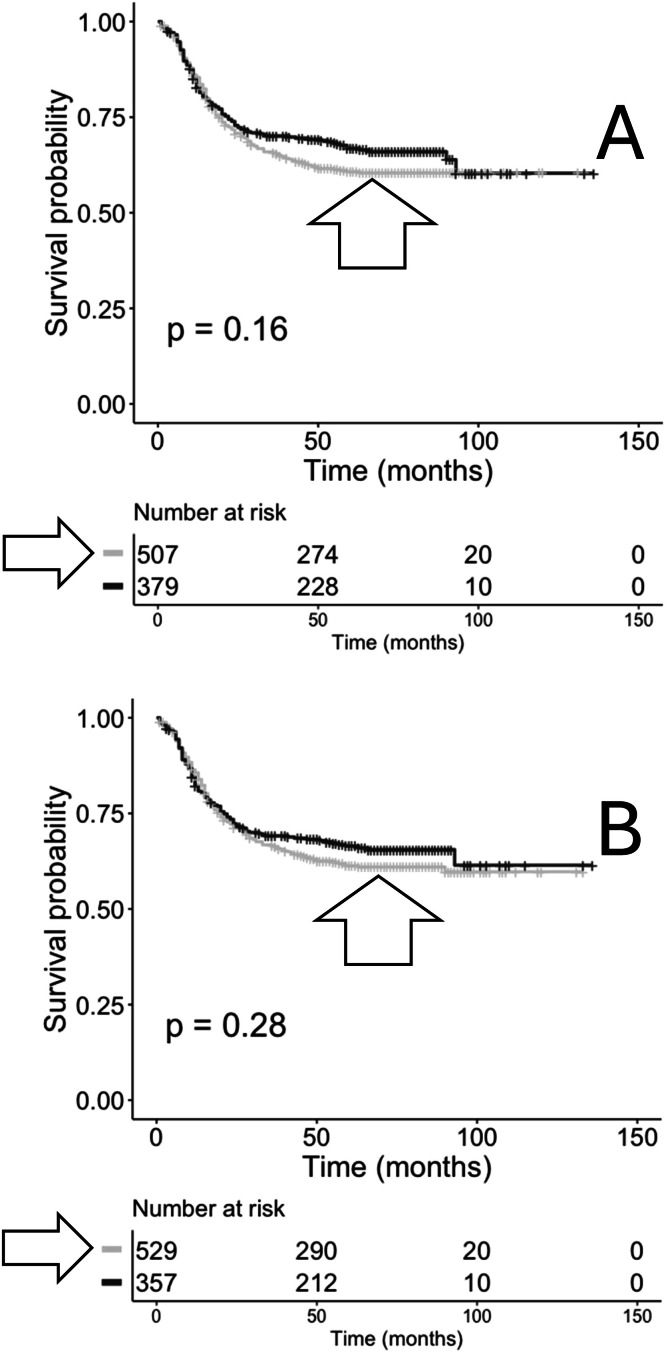
**Kaplan-Meier (KM) overall survival (OS) analyses of case IDs based on the recovery of TRA or TRB recombination reads from primary bone marrow samples.** (A) Case IDs with (grey, arrowhead, *n* = 507) or without (black, *n* = 379) TRA recombination reads. Log-rank *p* = 0.16. Hazard ratio: 0.8525. (B) Case IDs with (grey, arrowhead, *n* = 529) or without (black, *n* = 357) TRB recombination reads. Log-rank *p* = 0.28. Hazard ratio = 0.8824.

TRA and TRB recombination reads were extracted from the TARGET-AML RNAseq files (**Methods**). A total of 232,921 TRA and 409,757 TRB recombination reads were obtained, representing 2076 and 2116 unique cases (patients), respectively. An initial analysis representing only the primary bone marrow samples was performed to determine whether there were differences in OS probabilities based on the recovery, or lack of recovery of the TRA or TRB recombination reads. Results indicated that cases represented by recoveries of the TRA recombination reads indicated a trend of a lower OS probability in comparison to cases where no TRA recombination reads were recovered (Fig. 1A). In the case of TRB recombination read recoveries, there was no apparent OS probability distinction comparing recovery or lack of recovery of TRB recombination reads (Fig. 1B).

**Fig. 2:**
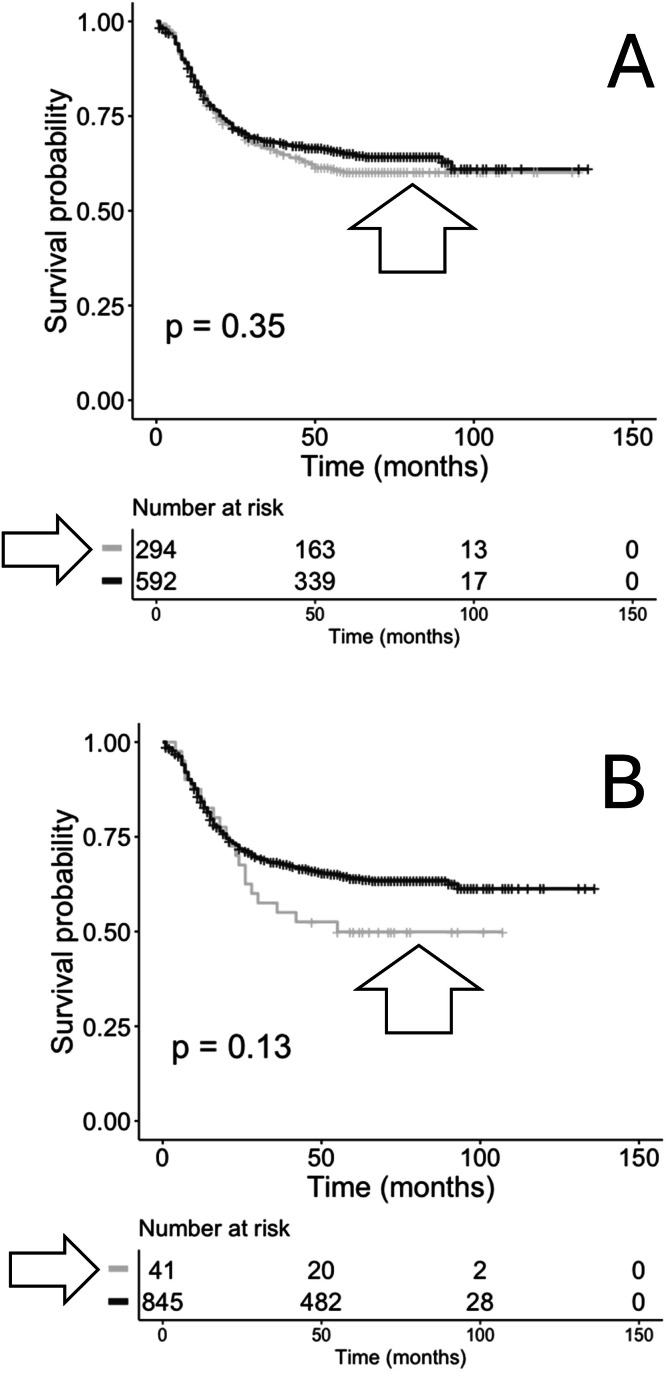
**Kaplan-Meier (KM) overall survival (OS) analyses of case IDs based on the recovery of TRA recombination reads, with or without matches to anti-CMV TRA CDR3s, from primary bone marrow samples.** (A) Case IDs with anti-CMV TRA CDR3s (grey, arrowhead; *n* = 294) or without anti-CMV TRA CDR3s (black; *n* = 592). Log-rank *p* = 0.35. Hazard ratio: 0.8956. (B) Repeat of (A), except the anti-CMV CDR3s were filtered for quality score of 1–3 before matching to the TRA recombination read CDR3s obtained from the AML primary bone marrow samples (grey, arrowhead; median survival=55 months; *n* = 41) and (black, median survival=not applicable; *n* = 845). Log-rank p-value = 0.13. Hazard ratio = 0.7048.

The CDR3 AA sequences represented by TRA recombination reads recovered from primary bone marrow samples were filtered for exact matches to a list of anti-CMV TRA CDR3 AA sequences obtained from the VDJdb (vdjdb.cdr3.net) [6]. Then, the cases representing TRA CDR3s with an exact AA sequence match to anti-CMV TRA CDR3s were assessed for OS. Initial analysis did not yield a significant difference in OS (Fig. 2A). The anti-CMV TRA CDR3s are scored on a scale of 0 to 3 with increasing confidence, as assessed by the VDJdb [6]. Thus, when anti-CMV TRA CDR3s were filtered to a score of 1 to 3 for matching to the AML primary bone marrow CDR3s, a decrease in OS probability was observed for the cases representing the anti-CMV TRA CDR3s, although the decrease represented a trend rather than statistical significance (Fig. 2B).

**Fig. 3:**
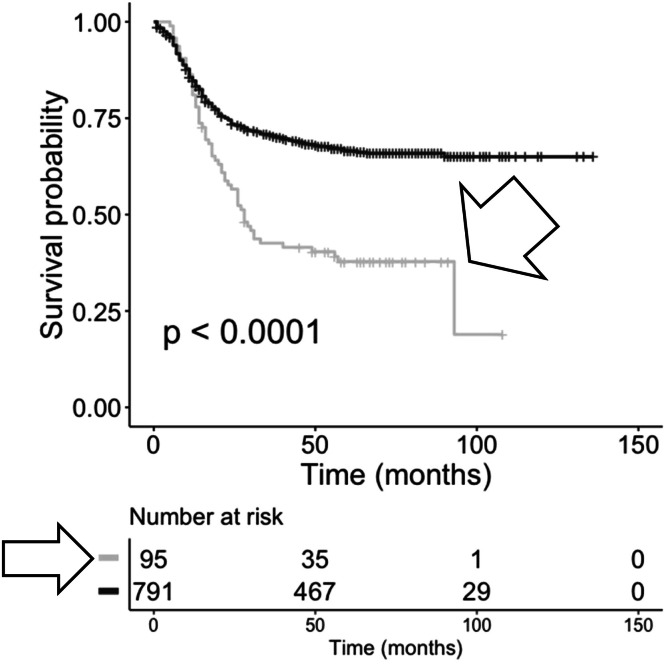
**KM OS analysis of case IDs based on the recovery of TRA recombination reads, with or without matches to anti-CMV TRA CDR3s from bone marrow samples representing recurrent AML.** Case IDs with anti-CMV TRA CDR3s (grey, arrowhead, median survival = 28 months, *n* = 95) and without anti-CMV TRA CDR3s (black, median survival, not applicable, *n* = 791). Log-rank p-value = <0.0001. Hazard ratio = 0.4676.

**Fig. 4:**
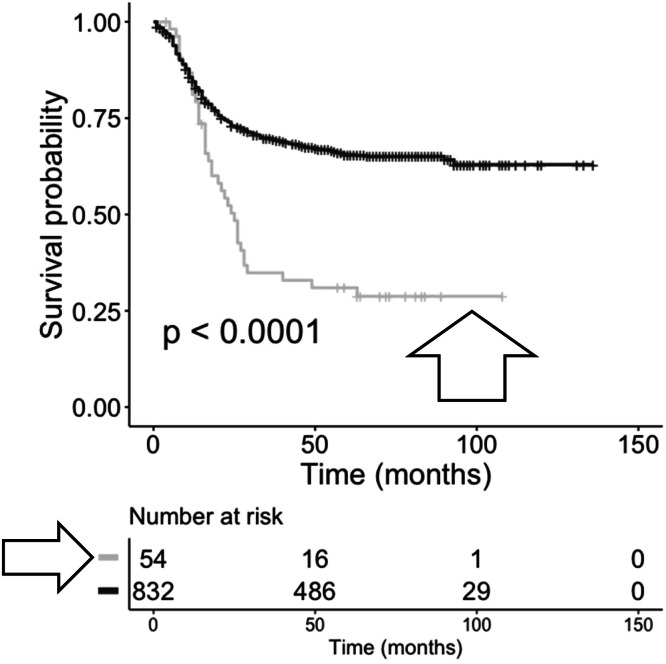
**KM OS analysis of case IDs based on the recovery of TRB recombination reads, with or without matches to anti-CMV TRB CDR3s from bone marrow samples representing recurrent AML.** Case IDs with anti-CMV TRB CDR3s (grey, arrowhead, *n* = 54) and without anti-CMV TRB CDR3s (black, *n* = 832). Log-rank p-value = <0.0001. Hazard ratio = 0.4124.

CDR3 AA sequences represented by TRA recombination reads recovered from bone marrow samples in recurrent AML were similarly filtered for exact matches to anti-CMV TRA CDR3 AA sequences from VDJdb. Cases representing exact matches to anti-CMV TRA CDR3s displayed a significant decrease in OS probabilities (Fig. 3; log-rank *p*= <0.0001). Similar analysis was performed on CDR3 AA sequences represented by TRB recombination reads using the anti-CMV TRB CDR3 AA sequences from VDJdb. Cases representing TRB CDR3 AA recombination reads with exact matches to anti-CMV TRB CDR3 AA sequences displayed a statistically significant reduction in OS probabilities (Fig. 4; log-rank *p*= <0.0001).

**Fig. 5:**
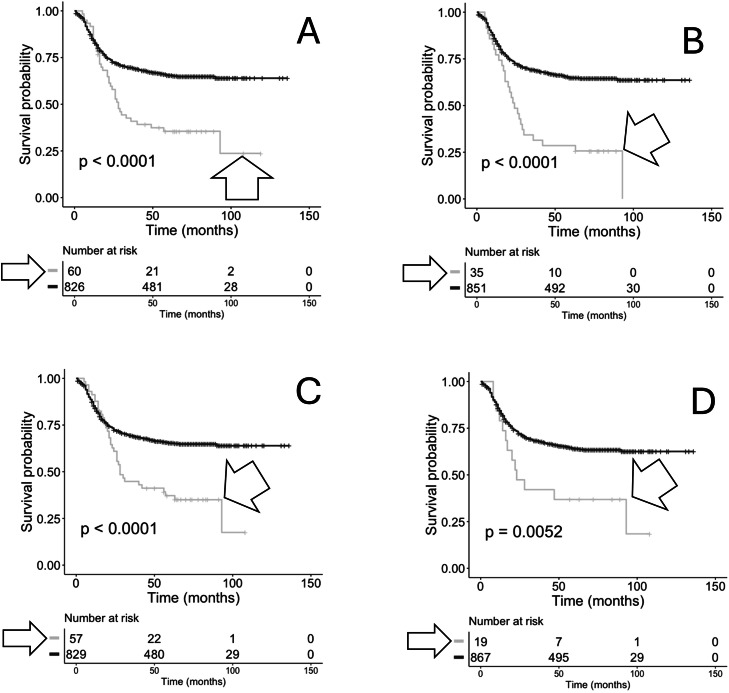
**KM OS analyses of case IDs based on the recovery of TRA and TRB recombination reads, with or without matches to anti-EBV or anti-Influenza TRA or TRB CDR3s from bone marrow samples representing recurrent AML.** (A) Case IDs with anti-EBV TRA CDR3s (grey, arrowhead, median survival = 28 months, *n* = 60) and without anti-EBV TRA CDR3s (black, media survival=not applicable, *n* = 826). Log-rank p-value = <0.0001. Hazard ratio = 0.4767. (B) Case IDs with anti-EBV TRB CDR3s (grey, arrowhead, median survival = 24 months, *n* = 35) and without anti-EBV TRB CDR3s (black, median survival=not applicable, *n* = 851). Log-rank p-value = <0.0001. Hazard ratio = 0.3752. (C) Case IDs with anti-Influenza TRA CDR3s (grey, arrowhead, median survival = 28 months, *n* = 57) and without anti-Influenza TRA CDR3s (black, median survival=not applicable, *n* = 829). Log-rank p-value = <0.0001. Hazard ratio = 0.4968. (D) Case IDs with and without TRB and anti-Influenza A CDR3s (grey, arrowhead, median survival = 23 months, *n* = 19) and without anti-Influenza TRA CDR3s (black median survival=not applicable, *n* = 867). Log-rank p-value = 0.0052. Hazard ratio = 0.5924.

The above analyses were extended to two additional sets of anti-viral CDR3s, anti-viral CDR3 AA sequences representing Epstein-Barr virus (EBV) and Influenza A (INFA). Recombination reads representing TRA from bone marrow samples representing recurrent AML cases were filtered for exact matches to anti-EBV TRA CDR3 AA sequences. Cases with anti-EBV TRA CDR3 AA sequences showed a significant reduction in OS probability (Fig. 5A; log-rank *p*= <0.0001). Similarly, TRB recombination reads with exact matches to anti-EBV TRB CDR3 AA sequences also showed a significant decrease in OS probability (Fig. 5B; log-rank *p*= <0.0001). Cases with anti-INFA TRA CDR3 AA sequences showed a significant reduction in OS probability (Fig. 5C; log-rank *p*= <0.0001). Similarly, TRB recombination reads with exact matches to anti-INFA TRB CDR3 AA sequences also showed a significant decrease in OS probability (Fig. 5D; log-rank *p*= <0.0052).

Multivariate analyses were performed to assess other clinical variables that may correlate with OS. TRA recombination reads from recurrent bone marrow representing matching anti-CMV CDR3s, age of diagnosis, patients identifying as Black or African American, risk group, and white blood cell count are all independently, significantly correlated with a reduction in OS (Table 1). The same results were obtained for a multivariate analysis with TRB recombination reads from recurrent bone marrow and matching anti-CMV CDR3s (Table 2).

**Table 1:** Multivariate analysis representing recurrent bone marrow samples from case IDs with a match for anti-CMV TRA CDR3s.

Characteristic	HR	95 % CI	p-value
Diagnosis Age	1.03	1.01, 1.05	0.002
WBC	1.00	1.00, 1.00 (1.00079, 1.0034)	0.003
Peripheral Blasts Percentage	1.00	1.00, 1.00	0.98
Sex			0.50
Female	—	—	
Male	0.92	0.73, 1.17	
Match or no match to anti-CMV CDR3s			<0.001
Match	—	—	
No Match	0.47	0.34, 0.64	
Race Category			<0.001
White	—	—	
Black or African American	2.20	1.63, 2.97	
Other	0.85	0.43, 1.67	
Asian	1.41	0.88, 2.27	
American Indian or Alaska Native	1.17	0.16, 8.44	
Native Hawaiian or other Pacific Islander	0.74	0.10, 5.34	
Risk Group			<0.001
Standard	—	—	
Low	0.35	0.26, 0.47	
High	1.23	0.90, 1.69	

Note: CMV, cytomegalovirus; CI, confidence interval; HR, Hazard Ratio; reference values for categorical variables are indicated by “—"; See Tables S7-S10 for further details.

**Table 2:** Multivariate analysis representing recurrent bone marrow samples from case IDs with a match for anti-CMV TRB CDR3s.

Characteristic	HR	95 % CI	p-value
Diagnosis Age	1.04	1.01, 1.06	<0.001
WBC	1.00	1.00, 1.00 (1.00083, 1.003)	0.003
Peripheral Blasts Percentage	1.00	1.00, 1.00	0.88
Sex			0.54
Female	—	—	
Male	0.93	0.73, 1.18	
Match or no match to anti-CMV CDR3s			<0.001
Match	—	—	
No Match	0.38	0.26, 0.56	
Race Category			<0.001
White	—	—	
Black or African American	1.42	0.88, 2.28	
Other	2.34	1.74, 3.16	
Asian	0.93	0.47, 1.82	
American Indian or Alaska Native	0.74	0.10, 5.30	
Native Hawaiian or other Pacific Islander	1.33	0.19, 9.61	
Risk Group			<0.001
Standard	—	—	
Low	0.34	0.25, 0.47	
High	1.26	0.92, 1.72	

Note: CMV, cytomegalovirus; CI, confidence interval; HR, Hazard Ratio; reference values for categorical variables are indicated by “—"; See Tables S7-S10 for further details.

## 4 Discussion

Primary bone marrow samples were a starting point for assessing OS probabilities for patients having anti-viral TCR CDR3s. In the case of anti-CMV TCR positive case IDs there was a lack of a statistically significant correlation with OS probabilities. In contrast, bone marrow samples representing recurrent disease provided an opportunity to observe a clear reduction in OS probabilities for anti-CMV positive case IDs, with replicability of approach, in that both TRA and TRB, anti-CMV CDR3s correlated with the reduced OS probability.

CMV is common in AML patients, although the precise relationship of CMV infections and patient outcomes can be variable and characterized by the stage of the cancer [15]. The findings in this report were replicated with anti-EBV and anti-Influenza A CDR3s representing both TRA and TRB. CMV and EBV, belonging to the same family, Herpesviridae, may have similar effects leading to the observed correlations with outcomes for AML patients. While these data do not provide a precise indication of the role of the virus in reducing OS probabilities, the data raise the questions of (a) whether detecting the anti-viral TCR CDR3s is a reflection of an infection as a comorbidity or (b) whether the detection of the anti-viral TCR CDR3s represents basically a status of high inflammation that, regardless of the specific pathogen, is inconsistent with a positive outcome?

A limitation throughout the analysis was the lack of comprehensive clinical data for all case IDs. Thus, with a more comprehensive collection of data, the described approaches could yield greater insights, especially when focusing on the higher quality scored anti-viral CDR3s, which was limited in this case by the lack of a bigger data collection. In sum, this study does support the idea that anti-viral sequences in TCR CDR3s representing viruses such as CMV and EBV correlate with disease progression in the case of recurrent AML. Potentially, our analyses could inspire a more careful CMV assessment in the case of recurrent AML and even possible application of ganciclovir, which has been successful in the glioblastoma setting [16].

## Funding

No funding to report.

## Ethics approval

The patient genomics files were accessed according to NIH dbGaP protocol approval number 16,405, representing application by George Blanck.

## Consent to participate

Administered by the TARGET project, phs000218.

## Consent for publication

Administered by the TARGET project, phs000218.

## CRediT authorship contribution statement

**Srijit Paul:** Conceptualization, Formal analysis, Methodology, Visualization, Writing – review & editing. **Michael T. Aboujaoude:** Conceptualization, Formal analysis, Methodology, Visualization, Writing – review & editing. **Vayda R. Barker:** Conceptualization, Formal analysis, Methodology, Visualization, Writing – review & editing. **Tabitha R. Hudock:** Conceptualization, Formal analysis, Methodology, Visualization, Writing – review & editing. **Arpan Sahoo:** Methodology, Software. **George Blanck:** Conceptualization, Formal analysis, Methodology, Project administration, Visualization, Writing – review & editing.

## Declaration of competing interest

Authors have nothing to declare.

## Appendix Supplementary materials

Image, application 1
